# Adsorption Behavior of C_10_ Amino Acid Surfactants at the Air/Water Interface: Implications for Membrane Compatibility

**DOI:** 10.1021/acs.langmuir.6c02078

**Published:** 2026-09-21

**Authors:** Diego R. Perinelli, Michele Verboni, Mattia Tiboni, Kun Ma, Peixun Li, Giulia Bonacucina, Marco Cespi, Simone Lucarini, Andrea Duranti, Giovanni Filippo Palmieri, Mario Campana, Luca Casettari

**Affiliations:** † Chemistry Interdisciplinary Project (ChIP), School of Pharmacy, 18959 University of Camerino, Camerino, Macerata 62032, Italy; ‡ Department of Biomolecular Sciences, School of Pharmacy, 19044 University of Urbino Carlo Bo, via Ca le Suore 2, Urbino, Pesaro e Urbino 61029, Italy; § ISIS Pulsed Neutron & Muon Source, 97008 STFC Rutherford Appleton Laboratory, Didcot OX110QX, U. K.

## Abstract

Amino acid-based surfactants represent a promising class of biocompatible and biodegradable amphiphiles with increasing relevance in pharmaceutical and biomedical applications. Despite their growing use, a quantitative understanding of their interfacial packing and how this relates to membrane interactions remains limited. Here, we combine surface tension measurements and neutron reflectivity (NR) to determine the area per molecule (APM), adsorbed amount (Γ), and interfacial structure of a series of C_10_-based *N*-acyl amino acid surfactants (C_10_-Leu, C_10_-Ser, and C_10_-Ala) at the air/buffer interface. NR reveals monolayers of comparable thickness (∼23.5 Å) for all surfactants, but with significant differences in packing density: C_10_-Leu exhibits the largest APM due to its bulky headgroup, whereas C_10_-Ser forms the most compact monolayer, likely stabilized by its hydroxyl-containing side chain. For C_10_-Ser and C_10_-Leu, APM values derived from NR were consistent with surface tension results, while NR resolved the uncertainty in the APM of C_10_-Ala from tensiometric measurements owing to its greater structural sensitivity. The interfacial packing characteristics correlate with the membrane behaviors previously observed within the C_10_ amino-acid surfactant series: surfactants with smaller APM and higher packing density (e.g., C_10_-Ser) were incorporated into lipid bilayers to a greater extent and were tolerated at higher concentrations before membrane disruption occurred, whereas surfactants with bulkier headgroups (e.g., C_10_-Leu) exhibited reduced incorporation and induced membrane disruption at lower concentrations. Measurements at 25 and 37 °C showed no significant temperature dependence. These findings demonstrate that interfacial packing provides a useful structural framework for rationalizing the membrane behavior of closely related amino acid-based surfactants, while highlighting that additional molecular factors are likely to contribute to surfactant–membrane interactions.

## Graphical abstract

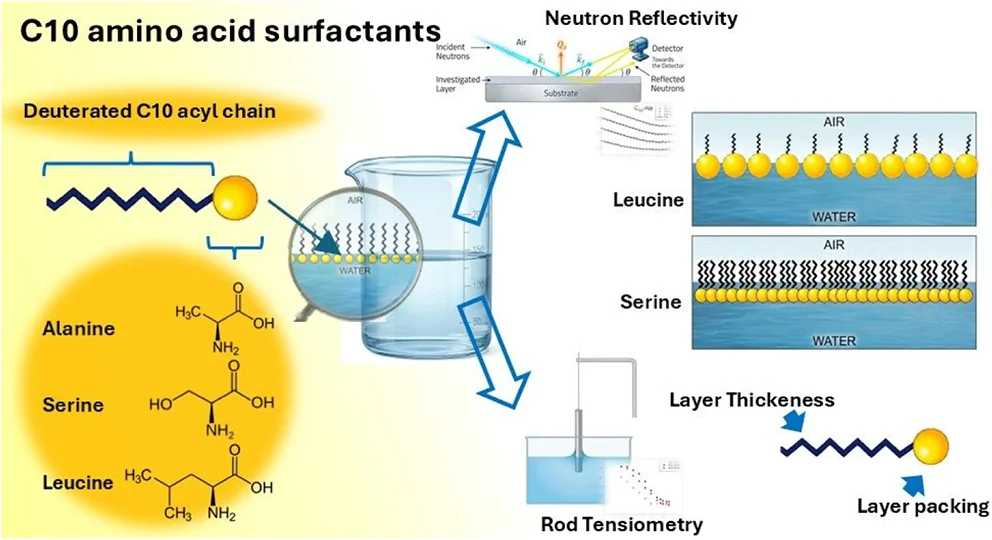

## Introduction

In recent years, amino acid-based surfactants have emerged as a promising class of biocompatible and biodegradable amphiphiles, attracting growing attention for their potential in pharmaceutical and biomedical applications.^1–3^ These anionic surfactants, commonly composed of a linear fatty acid tail and an amino acid derived polar headgroup, can combine structural versatility with environmental sustainability and a favorable toxicological profile.^2^ Their amphiphilic nature allows them to self-assemble in aqueous environments above a specific concentration, known as the critical micelle concentration (CMC), leading to the formation of a variety of nanoscale aggregates such as micelles, vesicles, and other supramolecular structures.^4^ While their role as mild detergents in cosmetic formulations is well established,^5^ amino acid-based surfactants are increasingly investigated for pharmaceutical and biomedical purposes, including drug solubilization, enhancement of membrane permeability, and the development of targeted nanocarriers for controlled drug delivery.^6–8^ However, to safely and effectively exploit their potential, a comprehensive understanding of their surface behavior and interaction with biological interfaces is essential.

Neutron reflectivity (NR) is a powerful, nondestructive technique that enables an accurate characterization of surfactant adsorption and structural organization at solid–liquid interfaces, such as model biological membranes.^9^ By providing nanoscale resolution of the interfacial composition, layer thickness, and hydration profiles, NR offers valuable insights into the surface aggregation behavior, adsorption kinetics, and potential membrane-disruptive effects of surfactants.^10,11^ This is particularly relevant for evaluating the biocompatibility and mechanistic interactions of amino acid–based surfactants with lipid bilayers or other biological surfaces.

In our previous study^12^ on the interactions between C_10_ amino-acid surfactants and DMPC bilayers, we demonstrated that all surfactants readily inserted into the membrane and exhibited comparable incorporation profiles up to concentrations approaching their respective CMCs. The principal difference emerged at higher surfactant concentrations. While bilayers exposed to C10-Ser and C10-Ala were able to accommodate progressively larger amounts of surfactant remaining intact up to approximately 3–4 times the CMC, C_10_-Leu, C_10_-Met and the remaining surfactants induced membrane disruption at approximately twice the CMC, accompanied by the formation of mixed micelles as confirmed by complementary SANS measurements. This behavior was attributed to the intrinsic interaction between the surfactants and the planar lipid bilayer. As the surfactant concentration within the membrane increases, the local curvature imposed by the surfactant can initially be accommodated by the bilayer; however, beyond a critical concentration, it eventually exceeds its ability to maintain a planar structure, leading to membrane disruption.

The motivation for the present work was therefore to investigate whether the intrinsic geometrical properties of this family of structurally related C_10_ amino-acid surfactants could provide a structural basis for the different membrane responses. We hypothesized that surfactants, whose preferred interfacial packing more closely resembles that of phospholipids, would be incorporated more readily into planar lipid bilayers and could therefore reach higher concentrations before membrane disruption occurs. Conversely, surfactants, with larger molecular areas and packing characteristics less compatible with phospholipid organization, would be expected to destabilize the bilayer at lower surfactant loadings.

Neutron reflectivity at the air/water interface provides a direct measurement of the interfacial adsorbed amount, from which the area per molecule (APM) can be determined with high accuracy. Combined with molecular dimensions and headgroup chemistry, this enables estimation of the surfactant packing parameter and provides a quantitative description of the preferred molecular packing at a planar interface. Although the air/water interface is not intended to replicate the complexity of a phospholipid bilayer, it provides a well-defined model system for comparing the intrinsic packing characteristics of closely related surfactants under identical experimental conditions. The area per molecule and packing parameter should therefore be regarded as descriptors of the preferred molecular organization at planar interfaces rather than sole predictors of membrane activity. Nevertheless, when comparing structurally analogous surfactants, these parameters provide a useful framework for rationalizing their relative ability to be accommodated within lipid bilayers before membrane disruption occurs.

## Experimental Section

### Materials

l-Amino acid methyl ester hydrochlorides, decanoic acid, and triethylamine (Et_3_N) were purchased from Fluorochem (Hadfield, UK). Sodium dodecyl sulfate d-25 (d-SDS) and decanoic acid d-19 were kindly provided by ISIS Deuteration Facility of Rutherford Appleton Laboratory (Didcot, UK). Oxalyl chloride [(COCl)_2_] was purchased from TCI (Zwijndrecht, Belgium). Hydrogenous Sodium Dodecyl Sulfate was purchased from Sigma (Milan, Italy) and purified by recrystallization 3 times in ethanol before use. D_2_O for the neutron reflectivity measurements was purchased from Sigma while milli-Q water was produced using an Elgastat water purifier. Dried sodium sulfate (Na_2_SO_4_) and all organic solvents were purchased from Sigma (Milan, Italy). Prior to use, dichloromethane (CH_2_Cl_2_) and chloroform (CHCl_3_) were dried with molecular sieves with an effective pore diameter of 4 Å. Disodium hydrogen phosphate (Na_2_HPO_4_), potassium dihydrogen phosphate (KH_2_PO_4_), sodium chloride (NaCl), and sodium hydroxide (KOH) were purchased from Carlo Erba (Milan, Italy). Isotonic PBS buffer at pH 7.4 was prepared by dissolving Na_2_HPO_4_ (22.2 mM), KH_2_PO_4_ (5.6 mM), and NaCl (123.3 mM) in a mixture of deionized H_2_O/D_2_O (918 mL/82 mL) for neutron reflectivity measurements or in H_2_O for the surface tension measurements.

### Chemistry

The synthesis of *N*-decanoyl-l-amino acid derivatives was carried out through a three steps procedure. First, the decanoyl chlorides (2a, b) were prepared from the corresponding decanoic acids (1a, b). Subsequently, the methyl ester hydrochlorides of alanine (3a), leucine (3b), and serine (3c) were coupled with the opportune acyl chloride to achieve the *N*-decanoyl-l-amino acid methyl ester compounds (4a–f). Finally, the basic hydrolysis of the latter afforded the desired *N*-decanoyl amino acids (5a–f). The same synthetic pathway was adopted for the formation of the *N*-deuterated decanoyl compounds starting from deuterated decanoic acid. The overall synthetic approach is reported in Figure 1.

**Figure 1:**
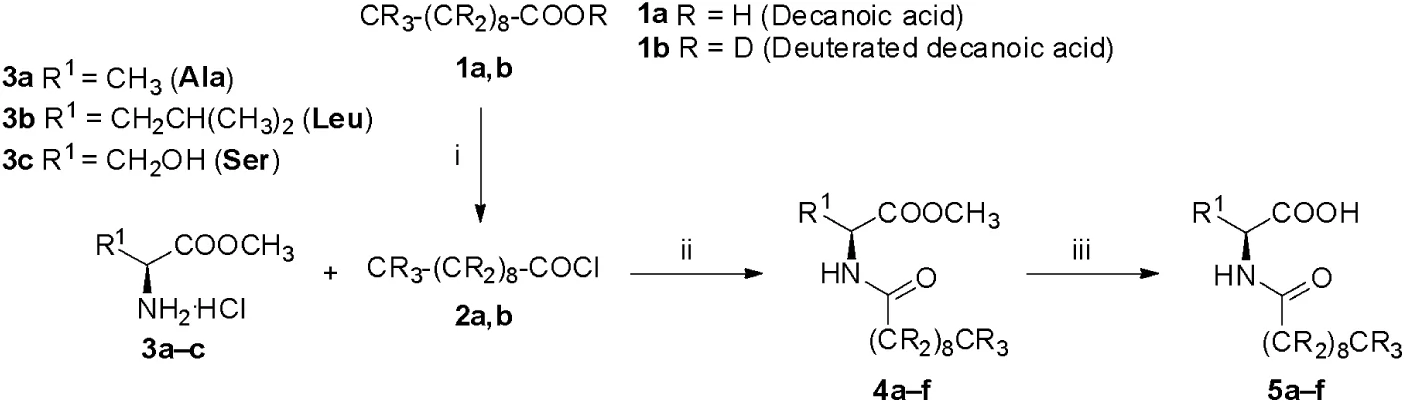
^a^Reagents and conditions: (i) (COCl)_2_, dry DMF, dry CH_2_Cl_2_, rt, 4 h; (ii) Et_3_N, dry CHCl_3_, 6 h; (iii) (a) KOH, THF/H_2_O 3:1, 1 h; (b) HCl/H_2_O, 0 °C.

#### General Procedure for the Synthesis of Decanoyl Chlorides (**2a, b**)

To a stirred solution of decanoic acid (**1a**) or deuterated decanoic acid (**1b**) (6.0 mmol) in dry CH_2_Cl_2_, oxalyl chloride (1.524 g, 1.03 mL, 12.0 mmol) and catalytic dry DMF were added, and the mixture was stirred at room temperature for 4 h. Then, the mixture was concentrated under reduced pressure to afford decanoyl chlorides (**2a, b**) as pale-yellow oils which were used without any further purification.

#### General Procedure for the Synthesis of *N*-Decanoyl Amino Acid Methyl Esters (**4a–f**)

To a solution of the opportune l-amino acid methyl ester hydrochloride (**3a–c**) (5.45 mmol) in dry CHCl_3_ (7.61 mL), Et_3_N (1.101 g, 1.53 mL, 10.9 mmol) was added and the mixture was stirred at 0 °C. Then, a solution of the opportune decanoyl chloride (**2a**,**2b)** in dry CHCl_3_ (3.05 mL) was added dropwise at the same temperature and the mixture was stirred at room temperature for 6 h. The mixture was diluted with CHCl_3_ and washed with NaHCO_3_ saturated solution, dried over ahnydrous Na_2_SO_4_, filtered, and concentrated under reduced pressure. The crude was purified by flash column chromatography (cyclohexane/EtOAc 8/2) to achieve **4a–f** as white solids.

*N-Decanoyl-* l *-alanine methyl ester (* **4a** *)*.^13^ Yield = 76%. MS (ESI): *m*/*z* = 258 [M + H]^+^. ^1^H NMR (CDCl_3_): δ = 0.88 (t, 3H, *J* = 7.0 Hz, CH_3_), 1.24–1.33 [m, 12H, (CH_2_)_6_], 1.41 (d, 3H, *J* = 7.0 Hz, CH_3_), 1.60–1.67 (m, 2H, COCH_2_ *CH*_2_), 2.21 (t, 2H, *J* = 7.5 Hz, CO*CH*_2_), 3.76 (s, 3H, OCH_3_), 4.58–4.66 (dq, 1H, *J*_1_ = *J*_2_ = 7.0 Hz, *CH*NH), 5.99 (br d, 1H, *J* = 7.0 Hz, NH) ppm. ^13^C NMR (CDCl_3_): δ = 14.1, 18.6, 22.6, 25.6, 29.19, 29.22, 29.3, 29.4, 31.8, 36.5, 47.8, 52.4, 172.6, 173.7 ppm.

*N-Decanoyl-* l *-leucine methyl ester (* **4b** *)*.^14^ Yield = 80%. MS (ESI): *m*/*z* = 300 [M + H]^+^. ^1^H NMR (CDCl_3_): δ = 0.85 (t, 3H, *J* = 7.0 Hz, CH_3_), 0.91 (d, 3H, *J* = 6.5 Hz, CH_3_), 0.92 (d, 3H, *J* = 6.0 Hz, CH_3_), 1.20–1.30 [m, 12H, (CH_2_)_6_], 1.46–1.55 [m, 1H, *CH*(CH_3_)_2_], 1.57–1.68 [m, 4H, *CH*_2_CH­(CH_3_)_2_, COCH_2_ *CH*_2_], 2.19 (t, 2H, *J* = 7.5 Hz, CO*CH*_2_), 3.70 (s, 3H, OCH_3_), 4.63 (ddd, 1H, *J*_1_ = 5.0 Hz, *J*_2_ = *J*_3_ = 9.0 Hz, *CH*NH), 5.97 (d, 1H, *J* = 9.0 Hz, NH) ppm. ^13^C NMR (CDCl_3_): δ = 14.2, 22.1, 22.8, 22.9, 25.0, 25.7, 29.33, 29.36, 29.44, 29.6, 32.0, 36.7; 41.9, 50.7, 52.3, 173.0, 173.9 ppm.

*N-Decanoyl-* l *-serine methyl ester (* **4c** *)*.^15^ Yield = 50%. MS (ESI): *m*/*z* = 274 [M + H]^+^. ^1^H NMR (CDCl_3_): δ = 0.88 (t, 3H, *J* = 7.0 Hz, CH_3_), 1.24–1.36 [m, 12H, (CH_2_)_6_], 1.63–1.69 (m, 2H, COCH_2_ *CH*_2_), 2.27 (t, 2H, *J* = 7.5 Hz, CO*CH*_2_), 2.55 (br t, 1H, *J* = 6.0 Hz, OH), 3.81 (s, 3H, OCH_3_), 3.91–4.01 (m, 2H, *CH*_2_OH) 4.70 (ddd, 1H, *J*_1_ = *J*_2_ = 3.5 Hz, *J*_3_ = 7.0 Hz, *CH*NH), 6.40 (d, 1H, *J* = 7.0 Hz, NH) ppm. ^13^C NMR (CDCl_3_): δ = 14.2, 22.8, 25.7, 29.37, 29.40, 29.5, 29.6, 32.0, 36.7, 52.9, 54.8, 63.8, 171.2, 173.9 ppm.

*N-D-decanoyl-* l *-alanine methyl ester (* **4d** *)*. Yield = 71%. MS (ESI): *m*/*z* = 277 [M + H]^+^. ^1^H NMR (CDCl_3_): δ = 1.41 (d, 3H, *J* = 7.0 Hz, CH_3_), 3.76 (s, 3H, OCH_3_), 4.62 (dq, 1H, *J*_1_ = *J*_2_ = 7.0 Hz, *CH*NH), 6.01 (d, 1H, *J* = 7.0 Hz, NH) ppm. ^13^C NMR (CDCl_3_): δ = 13.0 (septet, *J* = 18.5 Hz), 18.6, 21.6 (quintet, *J* = 19.0 Hz), 24.6 (quintet, *J* = 19.0 Hz), 27.8–28.4 (m, 4CD_2_), 30.5 (quintet, *J* = 19.0 Hz), 35.8 (quintet, *J* = 18.5 Hz), 47.9, 52.8, 172.9, 173.8 ppm.

*N-D-decanoyl-* l *-leucine methyl ester (* **4e** *)*. Yield = 72%. MS (ESI): *m*/*z* = 319 [M + H]^+^. ^1^H NMR (CDCl_3_): δ = 0.93 (d, 3H, *J* = 6.5 Hz, CH_3_), 0.95 (d, 3H, *J* = 6.0 Hz, CH_3_), 1.50–1.57 [m, 1H, *CH*(CH_3_)_2_], 1.60–1.68 [m, 2H, *CH*_2_CH­(CH_3_)_2_], 3.73 (s, 3H, OCH_3_), 4.66 (ddd, 1H, *J*_1_ = 5.0 Hz, *J*_2_ = *J*_3_ = 9.0 Hz, *CH*NH), 5.83 (d, 1H, *J* = 9.0 Hz, NH) ppm. ^13^C NMR (CDCl_3_): δ = 12.8 (septet, *J* = 19.0 Hz), 21.3 (quintet, *J* = 19.0 Hz), 21.9, 22.7, 24.5 (quintet, *J* = 19.0 Hz), 24.8, 27.4–28.4 (m, 4CD_2_), 30.4 (quintet, *J* = 18.5 Hz), 35.7 (quintet, *J* = 19.0 Hz), 41.7, 50.5, 52.1, 173.0, 173.8 ppm.

*N-D-decanoyl-* l *-serine methyl ester (* **4f** *)*. Yield = 45%. MS (ESI): *m*/*z* = 293 [M + H]^+^. ^1^H NMR (CDCl_3_): δ = 3.09 (br s, 1H, OH), 3.78 (s, 3H, OCH_3_), 3.88–3.98 (m, 2H, *CH*_2_OH), 4.67 (ddd, 1H, *J*_1_ = *J*_2_ = 3.5 Hz, *J*_3_ = 7.0 Hz, *CH*NH), 6.56 (d, 1H, *J* = 7.0 Hz, NH) ppm. ^13^C NMR (CDCl_3_): δ = 13.0 (septet, *J* = 18.0 Hz), 21.5 (quintet, *J* = 19.0 Hz), 24.6 (quintet, *J* = 19.0 Hz), 27.6–28.6 (m, 4CD_2_), 30.6 (quintet, *J* = 19.0 Hz), 35.8 (quintet, *J* = 18.5 Hz), 52.8, 54.7, 63.5, 171.2, 174.1 ppm.

#### General Procedure For the Synthesis of N-Decanoyl Amino Acids (**5a–f**)

To a stirred solution of the **4a–f** (2 mmol) in THF/H_2_O (3/1), potassium hydroxide (0.224 g, 4 mmol) was added and the mixture was stirred at room temperature for 1 h. Then, THF was removed under reduced pressure and the remaining aqueous solution was washed with ethyl acetate and treated with 10% HCl to pH 2. The resulting precipitate was extracted with ethyl acetate (2 × 20 mL) and the combined organic phases were washed with water, dried over anhydrous Na_2_SO_4_, filtrated, and concentrated under reduced pressure to obtain **5a–f** as white solids.

*N-Decanoyl-* *l* *-alanine* (**5a**). Yield = 90%. MS (ESI): *m*/*z* = 244 [M + H]^+^, 242 [M–H]^−^. ^1^H NMR (CDCl_3_): δ = 0.88 (t, 3H, *J* = 7.0 Hz, CH_3_), 1.24–1.33 [m, 12H, (CH_2_)_6_], 1.47 (d, 3H, *J* = 7.0 Hz, CH_3_), 1.60–1.67 (m, 2H, COCH_2_ *CH*_2_), 2.25 (t, 2H, *J* = 8.0 Hz, CO*CH*_2_), 4.58 (dq, 1H, *J*_1_ = *J*_2_ = 7.0 Hz, *CH*NH), 6.16 (d, 1H, *J* = 7.0 Hz, NH) ppm. ^13^C NMR (CDCl_3_): δ = 14.2, 18.1, 22.8, 25.7, 29.31, 29.39, 29.44, 29.6, 32.0, 36.6, 48.4, 174.2, 175.9 ppm.

*N-Decanoyl-* *l* *-leucine* (**5b**).^1^ Yield = 94%. MS (ESI): *m*/*z* = 286 [M + H]^+^, 284 [M–H]^−^. ^1^H NMR (CDCl_3_): δ = 0.88 (t, 3H, *J* = 7.0 Hz, CH_3_), 0.96 (d, 3H, *J* = 6.0 Hz, CH_3_), 0.97 (d, 3H, *J* = 6.5 Hz, CH_3_), 1.24–1.34 [m, 12H, (CH_2_)_6_], 1.54–1.68 (m, 3H, *CH*(CH_3_)_2_, COCH_2_ *CH*_2_), 1.69–1.77 (m, 2H, *CH*_2_CH­(CH_3_)_2_), 2.25 (t, 2H, *J* = 7.5 Hz, CO*CH*_2_), 4.62 (ddd, 1H, *J*_1_ = 5.0 Hz, *J*_2_ = *J*_3_ = 8.0 Hz, *CH*NH), 5.97 (br s, 1H, NH) ppm. ^13^C NMR (CDCl_3_): δ = 14.1, 21.9, 22.6, 22.8, 24.9, 25.6, 29.16, 29.22, 29.3, 29.4, 31.8, 36.5, 41.2, 50.8, 174.1, 176.4 ppm.

*N-Decanoyl-* *l* *-serine* (**5c**).^2^ Yield = 98%. MS (ESI): *m*/*z* = 258 [M–H]^−^. ^1^H NMR (DMSO-*d*_6_): δ = 0.86 (t, 3H, *J* = 6.5 Hz, CH_3_), 1.22–1.30 [m, 12H, (CH_2_)_6_], 1.44–1.51 (m, 2H, COCH_2_ *CH*_2_), 2.13 (t, 2H, *J* = 7.5 Hz, CO*CH*_2_), 3.58–3.69 (m, 2H, *CH*_2_OH), 4.26 (ddd, 1H, *J*_1_ = *J*_2_ = 4.5 Hz, *J*_3_ = 8.0 Hz, *CH*NH), 4.92 (br s, 1H, OH), 7.91 (d, 1H, *J* = 8.0 Hz, NH), 12.48 (br s, 1H, COOH) ppm. ^13^C NMR (DMSO-*d*_6_): δ = 14.4, 22.6, 25.7, 29.09, 29.14, 29.3, 29.4, 31.8, 35.5, 55.0, 61.9, 172.6, 172.8 ppm.

*N-* d-decanoyl- *l* *-alanine* (**5d**). Yield = 90%. MS (ESI): *m*/*z* = 263 [M + H]^+^, 261 [M–H]^−^. ^1^H NMR (CDCl_3_): δ = 1.47 (d, 3H, *J* = 7.0 Hz, CH_3_), 4.58 (dq, 1H, *J*_1_ = *J*_2_ = 7.0 Hz, *CH*NH), 6.06 (br s, 1H, NH) ppm. ^2^H NMR (CH_2_Cl_2_): δ_D_ = 0.78–0.98 (m, 3D, CD_3_), 1.13–1.46 [m, 12D, (CD_2_)_6_], 1.50–1.71 (m, 2D, COCD_2_ *CD*_2_), 2.11–2.40 (m, 2D, CO*CD*_2_) ppm. ^13^C NMR (CDCl_3_): δ = 13.0 (septet, *J* = 19.5 Hz), 18.3, 21.5 (quintet, *J* = 19.0 Hz), 24.6 (quintet, *J* = 19.5 Hz), 27.5–28.5 (m, 4CD_2_), 30.6 (quintet, *J* = 19.0 Hz), 35.7 (quintet, *J* = 19.5 Hz), 48.4, 174.4, 176.1 ppm.

*N-* d-decanoyl- *l* *-leucine* (**5e**). Yield = 94%. MS (ESI): *m*/*z* = 286 [M + H]^+^, 284 [M–H]^−^. ^1^H NMR (CDCl_3_): δ = 0.96 (d, 3H, *J* = 6.0 Hz, CH_3_), 0.97 (d, 3H, *J* = 6.5 Hz, CH_3_), 1.54–1.64 [m, 1H, *CH*(CH_3_)_2_], 1.65–1.77 [m, 2H, *CH*_2_CH­(CH_3_)_2_], 4.62 (ddd, 1H, *J*_1_ = 5.0 Hz, *J*_2_ = *J*_3_ = 8.0 Hz, *CH*NH), 5.99 (d, 1H, *J*_1_ = 8.0 Hz, NH) ppm. ^2^H NMR (CH_2_Cl_2_): δ_D_ = 0.78–0.89 (m, 3D, CD_3_), 1.11–1.34 [m, 12D, (CD_2_)_6_], 1.49–1.65 (m, 2D, COCD_2_ *CD*_2_), 2.08–2.31 (m, 2D, CO*CD*_2_) ppm. ^13^C NMR (CDCl_3_): δ = 12.9 (septet, *J* = 19.0 Hz), 21.3 (quintet, *J* = 19.0 Hz), 21.9, 22.8, 24.7 (quintet, *J* = 19.5 Hz), 24.9, 27.6–28.5 (m, 4CD_2_), 30.4 (quintet, *J* = 19.5 Hz), 35.6 (quintet, *J* = 19.5 Hz), 41.2, 50.8, 174.3, 176.4 ppm.

*N-* d-decanoyl- *l* *-serine* (**5f**). Yield = 95%. MS (ESI): *m*/*z* = 277 [M–H]^−^. ^1^H NMR (DMSO-*d*_6_): δ = 3.60 (dd, 1H, *J*_1_ = 4.5 Hz, *J*_2_ = 11.0 Hz, *CH*_*2a*_OH), 3.67 (dd, 1H, *J*_1_ = 5.5 Hz, *J*_2_ = 11.0 Hz *CH*_2b_OH), 4.26 (ddd, 1H, *J*_1_ = 4.5 Hz, *J*_2_ = 5.5 Hz, *J*_3_ = 8.0 Hz, *CH*NH), 4.97 (br s, 1H, OH), 7.89 (d, 1H, *J* = 8.0 Hz), 12.44 (br s, 1H, COOH) ppm. ^2^H NMR [DMSO]: δ_D_ = 1.29–1.47 (m, 3D, CD_3_), 1.61–2.12 [m, 14D, (CD_2_)_6_, COCD_2_ *CD*_2_], 2.43–2.91 (m, 2D, CO*CD*_2_) ppm. ^13^C NMR (DMSO-*d*_6_): δ = 13.0 (septet, *J* = 19.0 Hz), 21.2 (quintet, *J* = 19.0 Hz), 24.6 (quintet, *J* = 19.5 Hz), 27.5–28.4 (m, 4CD_2_), 30.3 (quintet, *J* = 19.0 Hz), 34.7 (quintet, *J* = 19.0 Hz), 55.0, 61.9, 172.6, 172.8 ppm.

The structures of compounds were unambiguously assessed by MS, ^1^H NMR, ^2^H NMR and ^13^C NMR. ESI-MS spectra were recorded with a Waters Micromass ZQ spectrometer in a negative or positive mode using a nebulizing nitrogen gas at 400 L/min and a temperature of 250 °C, cone flow 40 mL/min, capillary 3.5 kV and cone voltage 60 V; only molecular ions [M–H]^−^ or [M–H]^+^ are given. ^1^H NMR, ^2^H NMR, and ^13^C NMR spectra were recorded on a Bruker AC 400, 61.4 or 101, respectively, spectrometer and analyzed using the TopSpin software package. Chemical shifts were measured by using the central peak of the solvent and, in the case of ^2^H NMR, CDCl_3_ was used as internal standard. Column chromatography purifications were performed under “flash” conditions using Merck 230–400 mesh silica gel. TLC was carried out on Merck silica gel 60 F254 plates, which were visualized by exposure to an aqueous solution of potassium permanganate.

The *N*-decanoyl amino acid surfactants were synthesized in the acid form. For the further characterization in the dispersion state, the surfactants were dissolved in a phosphate/NaCl buffer. Upon dissolution, their carboxylic acid groups are expected to be deprotonated. Therefore, they are negatively charged under measurement conditions, with counterions supplied by the buffer. The buffer ensures that they are charged and soluble in aqueous medium, otherwise insoluble.

### Surface Tension Measurements

Surface tension measurements were performed using a Kruss K11 maximum pull tensiometer. Because of the limited availability of deuterated surfactant, measurements were performed using a platinum rod with known geometry instead of the standard plate. The amount of SDS d-25 was very limited; therefore, surface tension measurements for SDS were carried out using h-surfactant. The rod was rinsed in Milli-Q water and dried using a Bunsen burner prior to each measurement. The tensiometer was calibrated in Milli-Q water first, where a surface tension of 72 mN m^–1^ was achieved. All subsequent measurements were performed in a phosphate buffer. Measurements were performed in triplicates. Low-molecular-weight surfactants generally attain equilibrium surface tension on relatively short time scales (typically seconds to minutes), although the adsorption kinetics depend on surfactant type and may be slowed for ionic surfactants in the absence of added electrolyte. Surface tension was therefore measured in kinetic mode: measurements were acquired every 6 s for up to 1 h, or until the instrument’s termination criterion was met. Measurements were terminated automatically when the standard deviation of the previous 10 consecutive surface tension values was ≤0.05 mN m^–1^, at which point the surface tension was considered to have reached equilibrium. The temperature was maintained to 37 ± 1 °C by means of a circulating water bath. The surface excess at the interface was calculated using the Gibbs adsorption equation:

1 $$\Gamma =-\frac{1}{mRT}\frac{d\gamma }{d\ln C}$$

Where d*γ/*d ln *C* is the variation of interfacial tension as a function of logarithm of concentration, *R* is the gas constant, *T* is the temperature, and *m* is a prefactor dependent on how many species contribute to the surface chemical potential change, i.e., on which counterions are associated with the adsorbed surfactant. d*γ/d* ln *C* was determined by linear least-squares regression. Standard errors of the fitted slopes were obtained from the regression analysis and propagated to calculate the uncertainties in the derived parameters using standard error propagation. Strictly, the Gibbs adsorption equation is formulated in terms of the mean ionic activity rather than the analytical concentration. However, under the experimental conditions employed in this work, the ionic strength is largely dominated by the background electrolyte, resulting in a calculated variation of the mean ionic activity coefficient of surfactant of less than 1% over the concentration range investigated. Consequently, the mean ionic activity is effectively proportional to the analytical surfactant concentration, and the latter was therefore used throughout the analysis. The surfactants investigated in this study are available as sodium salts, therefore in principle a value of m = 2 should be used.^16^ However, counterions are approximately constant as they come from buffer in excess, therefore the use of m = 1 is more appropriate in these systems. The critical micelle concentration (CMC) was determined from the breakpoint in the surface tension versus logarithm of the surfactant concentration plot. The CMC was taken as the intersection of linear regressions fitted to the pre-CMC region, where surface tension decreased with increasing surfactant concentration, and the post-CMC region, where the surface tension was approximately constant

### Neutron Reflectivity

The neutron reflectivity measurements were performed at the Surf reflectometer at ISIS, Didcot, RAL.^17,18^ The polychromatic beam, with wavelength λ between 0.5 and 7 Å, enables to measure the reflectivity over a broad *Q* range, where *Q* is the wave vector transfer perpendicular to the surface defined as

2 $$Q=\frac{4\pi \sin\sin\;\theta }{\lambda }$$

θ is the incident angle, which was fixed at 1.5°, covering a Q range between 0.048 Å^–1^ and 0.5 Å^–1^.

The reflectivity profiles were analyzed using RasCAL. The interface is divided in a finite number of layers, each layer characterized by a certain thickness (d), roughness (σ) and scattering length density (ρ). The scattering length density of a layer is the sum of the scattering length density of each component within the layer and is a function of layer composition:

3 $$\rho_{Layer}=\underset{i}{\sum }\Phi_{i}\rho_{i}$$

Φ and ρ are the volume fraction and scattering length density of every component in the layer, respectively. The reflectivity is calculated from the model using the optical matrix method^19^ and compared to the experimental data. The fitting routine is then iterated to achieve a least-squares minimization.

All measurements were performed using a buffer composed of ∼92% H_2_O and ∼8% D_2_O, referred to as null reflecting water (NRW). Chain-deuterated surfactants were employed to maximize the neutron scattering contrast between the adsorbed layer and the aqueous subphase. The resulting increase in contrast enhances the sensitivity of neutron reflectometry to the structure and composition of the surfactant layer at the air/water interface. With this contrast the reflectivity arises selectively from the interfacial layer and the reflectivity profile delivers information on thickness and scattering length density of the adsorbed layer, which can then be used to calculate the surfactant adsorbed amount:^20^

4 $$\Gamma =\frac{{\left(\rho \right)}_{layer}}{\Sigma_{i}b_{i}}\frac{d}{N_{A}}$$

Where *d* and *(ρ)*_*layer*_ are the experimentally determined thickness and scattering length density of the layer, respectively. All reflectivity profiles were fitted using a single layer with no interfacial roughness. Although surfactant monolayers may be represented by separate headgroup and tail-group layers, the contrast between the hydrogenous headgroup region and the aqueous subphase is typically low. Combined with the significant hydration of the headgroup region, it substantially reduces the sensitivity of the reflectivity data to its structural parameters. Consequently, the contribution of a distinct headgroup layer is often marginal, and a single-layer model provides an adequate and robust description of the adsorbed surfactant layer. The surfactants measured in this study were SDS, C_10_-Leu, C_10_-Ala and C_10_-Ser. The values for scattering length *b* are given in Table 1 for all surfactants; exchange of labile hydrogens with the bulk phase has been considered where required. The area per molecule, APM, is then calculated from the equation:

5 $$\mathrm{APM}=\frac{10^{20}}{\Gamma N_{A}}$$

**Table 1:** Scattering Length for the Chain Deuterated Surfactants Investigated in This Study

Surfactant	Scattering length *b*/fm molec^–1^
C_10_-Leu	243.93
C_10_-Ala	227.72
C_10_-Ser	224.78
SDS	271.06

The uncertainty in the fitting parameters was obtained using Bayesian probability routines available with RasCAL. In the text every fitted parameter will be referred to as the closest half integer to the optimum fit, detailed values are displayed in the relevant tables.

The surfactant solutions were poured in 5 custom-built stainless-steel troughs coated with a thin PTFE sheet. The overall required volume is around 45 mL, decreasing to ∼25 mL for surfactant solution at high concentration because of improved wettability. Measurements were performed at 25 °C and at 37 °C, constant temperature was maintained using a circulating water bath running underneath each individual trough. Every trough was encased in a box, the temperature of which was maintained at 1 °C higher than the solution using electric heating mats to avoid condensation. Thirty minutes of equilibration was allowed when changing temperature. Measurements were taken at several concentrations below and above the CMC with the aim to describe the adsorption isotherm and determine the surfactant area per molecule (APM).

## Results and Discussion

### Surface Tension

Surface tension was not only measured for all the chain-deuterated C_10_-surfactants (Figure 2A) but also repeated on freshly prepared batches of hydrogenous surfactants for comparison,^12^ results are shown in Figure 2B. Surface tension data for SDS are shown in Figure S1 in Supporting Information. The Area Per Molecule (APM) for all surfactants as calculated from eq 1 is shown in Table 2. The surface tension profile for d-C_10_-Leu seems to be shifted to lower concentration values compared to the correspondent h-C_10_-Leu, but the slope, hence APM and surface excess, are nearly identical between the two sets. The resulting APM for d- and h-C_10_-Leu and C_10_-Ser are within error and agree very well with results reported previously.^21,22^ The CMC for d-C_10_-Leu was found to be moderately lower compared to the fully hydrogenous surfactant (∼2.5 mM for the d-surfactant, ∼5 mM for the h-surfactant). As surface-active impurities would be expected to influence both the slope (dγ/d ln *C*) and the shape of the surface tension curve around the CMC, the observed discrepancy is unlikely to arise from such impurities and may instead reflect the presence of nonsurface-active impurities. Unfortunately, no additional tests were possible on the material due to limited availability. However, the slope in the two surface tension profiles, hence the parameter dγ/d ln *C* in eq 1, was within error: as a result, the calculated APM was not affected by this discrepancy. On the other hand, results for C_10_-Ala showed a much larger degree of variation. Results are in close agreement between the h- and d-surfactants but note how the APM for chain-deuterated C_10_-Ala has a much larger error bar associated. This may be attributed to the lack of a sharp change in the surface tension profile around the CMC, which is present instead with the h-surfactant. The APM is also much lower compared to the results previously reported for C_10_-Ala under the same experimental conditions (55.6 ± 2.0 Å^2^).^21^ The necessity for an independent determination of APM from neutron reflectivity arises precisely from this discrepancy, which renders these measurements potentially unreliable and hinders the understanding of their previously reported behavior in the presence of lipid bilayers.^12^

**Figure 2:**
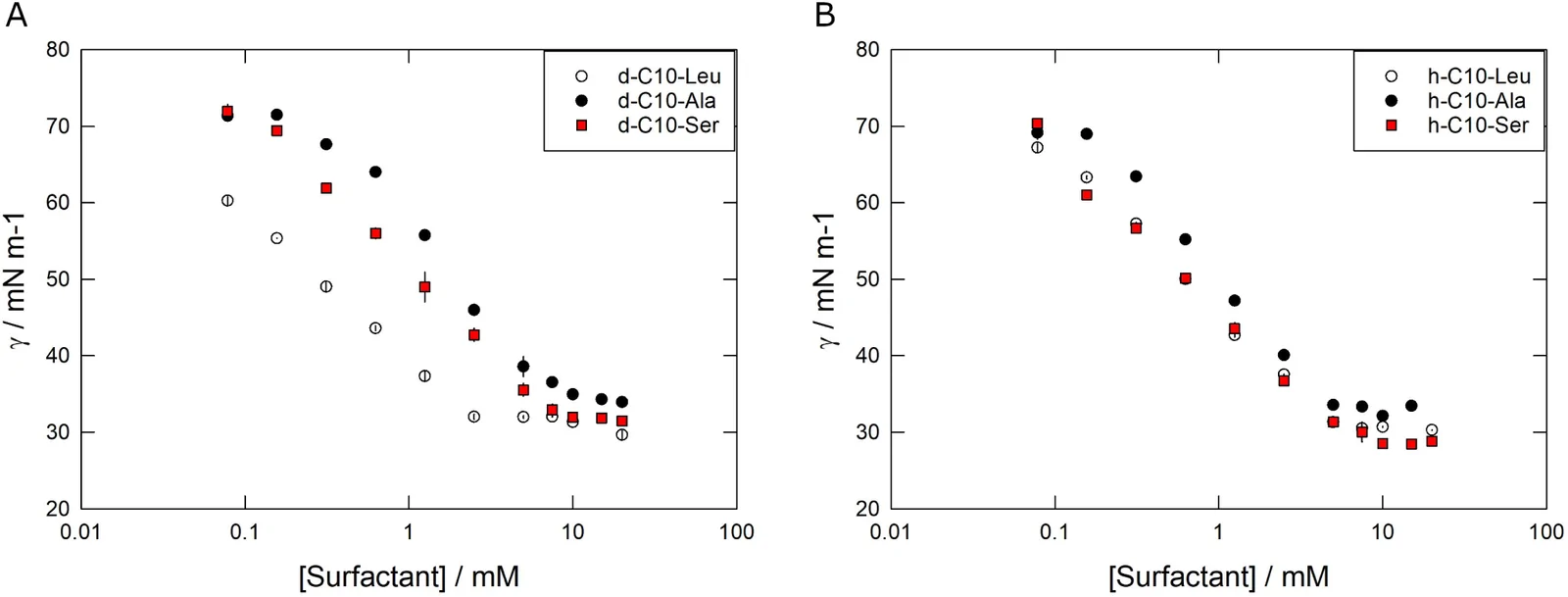
Surface tension of C_10_-Leu, C_10_-Ala, and C_10_-Ser with the deuterated chain (A) and hydrogenous chain (B). For C10-Leu we observed a lower CMC for the deuterated version.

**Table 2:** Area per Molecule (APM) at Air/Water Interface for All Surfactants Studied from Neutron Reflectivity (NR) and Surface Tension measurements.^a^

	Surface tension	Neutron Reflectivity	
Surfactant	APM (ST)/Å^2^	APM (NR)/Å^2^	Γ(NR) μmol m^–2^
C_10_-Leu	51.7± 2.1 (d); 53.3 ± 3.9 (h)	48.2 (46.4–50.4)	3.44 (3.31–3.59)
C_10_-Ala	48.4 ± 7.0 (d); 39.6 ± 2.1 (h)	43.0 (41.4–45.0)	3.86 (3.71–4.02)
C_10_-Ser	44.9 ± 2.3 (d); 45.7 ± 1.7 (h)	38.7 (37.2–40.5)	4.29 (4.12–4.47)
SDS	47.2 ± 2.4 (h)	33.7 (32.7–34.8)	4.92 (4.78–5.08)

a The maximum adsorbed amount (Γ), as determined from NR, is also reported. The C10-aa surfactants were measured at 20 mM, SDS at 5 mM. Full results for all concentrations are presented in Figure 4 with all values listed in Table S1.

### Neutron Reflectivity

All reflectivity profiles for the C_10_-aa surfactants are shown in Figure 3 along with the best line fit. Results for Area Per Molecule (APM) and Adsorbed amount (Γ) are shown in Table 2 for all surfactants. Only results at 37 °C are reported here as no measurable differences were observed lowering the temperature to 25 °C.

**Figure 3:**
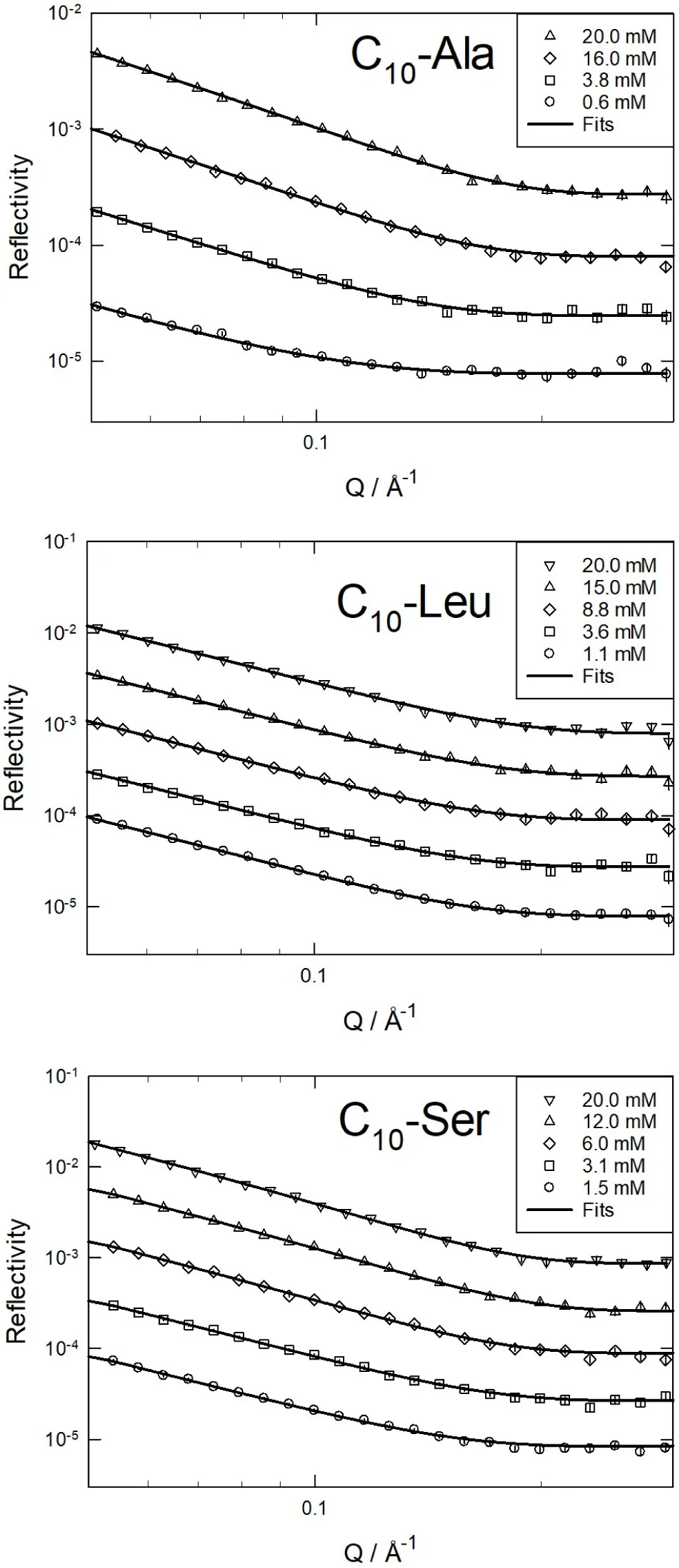
Reflectivity profiles for C_10_-Ala, C_10_-Ser, and C_10_-Leu at different concentrations measured at the air/NRW interface at 37 °C. Best line fits are superimposed with the data points, and profiles are shifted in reflectivity for clarity of presentation.

SDS has been widely characterized at the air/water interface using neutron reflectivity and much is known about this system. However, the only characterization of interfacial properties under buffer conditions to our knowledge dates back several decades,^23^ and some studies were performed under different experimental (black film) conditions.^24,25^ In a study by Lu et al. SDS was studied at the air/water interface in pure water and the APM was found to be around 47.4 Å^2^.^26^ Under buffer conditions one would expect the head-to-head repulsion between the SDS sulfate groups to be much smaller compared to pure aqueous solution, thus allowing a tighter packing between the molecules, with a smaller APM and higher Γ. This was indeed the case: the area per molecule was found to be around 33.7 Å^2^, much smaller than the 47.4 Å^2^ in pure water;^26^ this clearly reflects in a higher adsorbed amount Γ at the interface (∼4.92 μmol m^–2^ compared to 3.50 μmol m^–2^). These values are in good agreement with the values reported by Nilsson et al. and Belorgey and Benattar^23,25^ The fitted layer thickness at full coverage was 22.5 ± 1 Å, slightly higher than the 18 Å value reported at the air/water interface. This may be due to more staggering within the monolayer, possibly because of the reduced lateral repulsion, which allows to accommodate the excess material.

For all three C_10_-aa surfactants studied the layer thickness at full coverage was about 23.5 ± 2 Å, with very little difference between them. This value is slightly higher than SDS, which has a longer hydrocarbon chain, thus indicating a higher disorder and possibly more staggering within the adsorbed monolayer. Of the three C_10_-aa surfactants characterized in this experiment, leucine is the amino acid with the largest headgroup as it has a bulkier side chain, thus it is not surprising that its derivative is the surfactant with the largest APM in the series. Serine and alanine have strikingly similar molecular volumes^27^ but C_10_-Ser has a ∼10% smaller APM. This difference is likely attributable to the presence of the hydroxyl group in the serine side chain, which may promote a more favorable orientation of the headgroups within the monolayer and facilitate a more efficient molecular packing. These observations suggest that, in addition to steric considerations, headgroup chemistry—including polarity, hydration, and hydrogen-bonding capability—plays an important role in determining the interfacial packing of these surfactants.

The shaded areas in Figure 4 show the surface excess determined from surface tension and enable a visual comparison with the neutron reflectivity results. For C_10_-Leu the APM as determined from neutron reflectivity is in excellent agreement with that calculated from the surface tension isotherms, while for C_10_-Ser it falls just within the error bars. While the value for APM for C_10_-Ala falls within the acceptance range for the surface tension data, it is noteworthy that this range had a large uncertainty associated. Since NR provides a much higher sensitivity to interfacially adsorbed layers, the NR data enables to determine the value of APM of the adsorbed surfactant with a much higher degree of confidence, hence enabling the discussion of the physical phenomena observed during the interaction with lipid bilayers.^12^ Notably, results for SDS deviate significantly between the two techniques. The CMC of SDS in the phosphate/NaCl buffer is approximately 1 mM, substantially lower than the value of ∼8 mM reported for pure water, reflecting the screening of electrostatic repulsion between adjacent sulfate headgroups by the high ionic strength of the buffer. Such electrostatic screening is expected to promote closer molecular packing at the interface and, consequently, a smaller area per molecule than is typically reported for SDS at the air–water interface in pure water. This trend is consistent with the neutron reflectivity measurements, whereas the area per molecule derived from the Gibbs adsorption analysis remains comparable to literature values reported for pure water.^26,28^ The origin of this discrepancy is not clear and was not investigated further in the present study. It should also be noted that neutron reflectivity provides a direct structural determination of the interfacial adsorbed amount through modeling of the scattering-length-density profile. In this respect, the neutron reflectivity results are considered to provide a more direct measure of the interfacial molecular packing.

**Figure 4:**
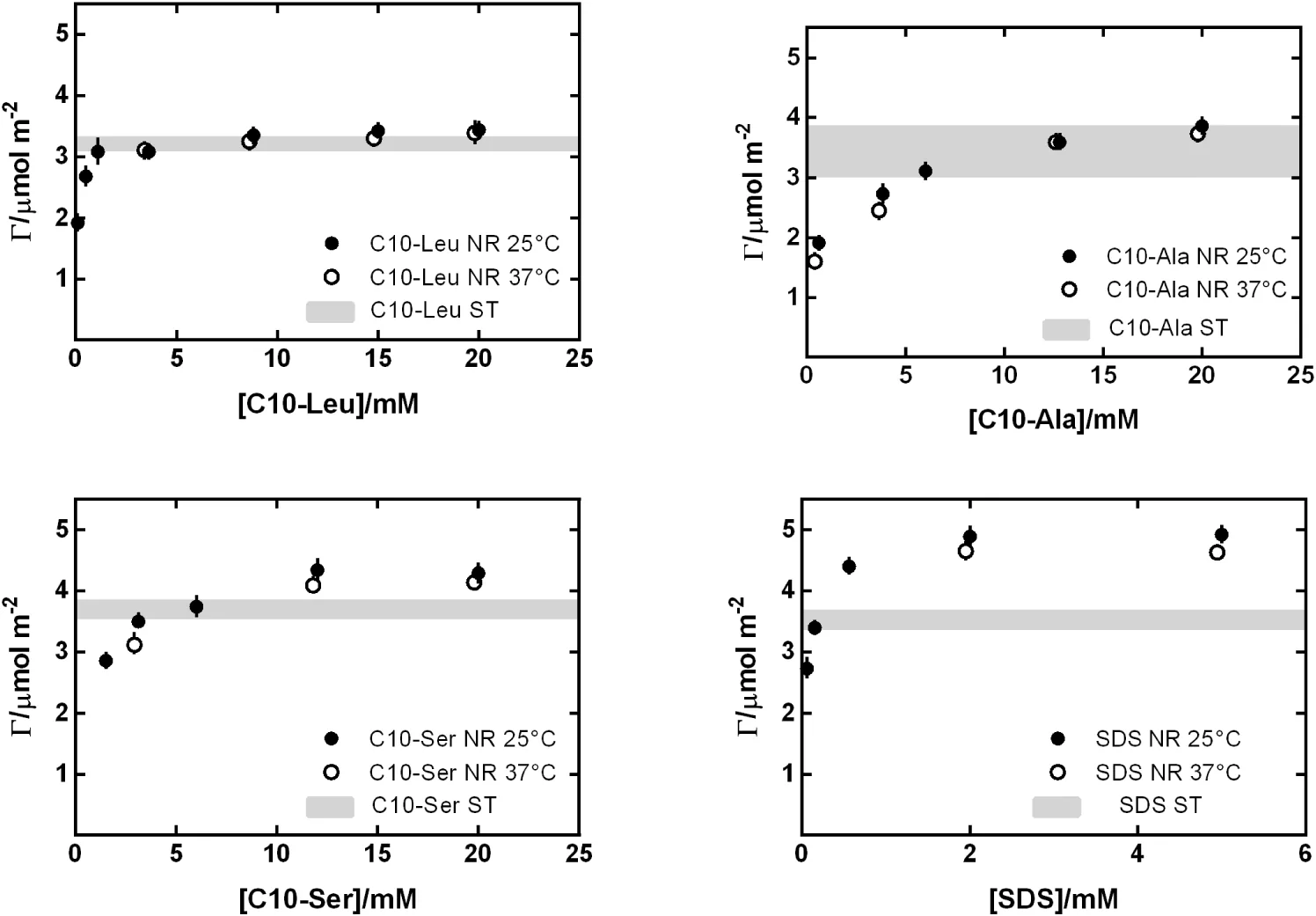
Adsorbed amount Γ at the air/NRW interface for all surfactants (C_10_-Leu, C_10_-Ala, C_10_-Ser, and SDS) at different concentrations studied at 25 °C (black dot) and 37 °C (white circle) compared to the Γ value determined from surface tension results at the CMC (gray box in each plot).

The reason for performing measurements both at 25 and 37 °C is that experiments are often reported at room temperature instead of bodily temperature, therefore it is vital to verify if the adsorption profile differs under these conditions. Because of the longer equilibration time at higher temperature, more data points were performed at 25 °C, resulting in more detailed isotherms, as shown in Figure 4. When measurements are performed at higher temperature, the higher thermal energy may overcome the electrostatic repulsion between charged headgroups, potentially allowing closer packing at the interface, hence larger Γ. In our study, the different temperature was not sufficiently large to produce measurable differences (Figure 5), therefore the effect can be considered negligible.

**Figure 5:**
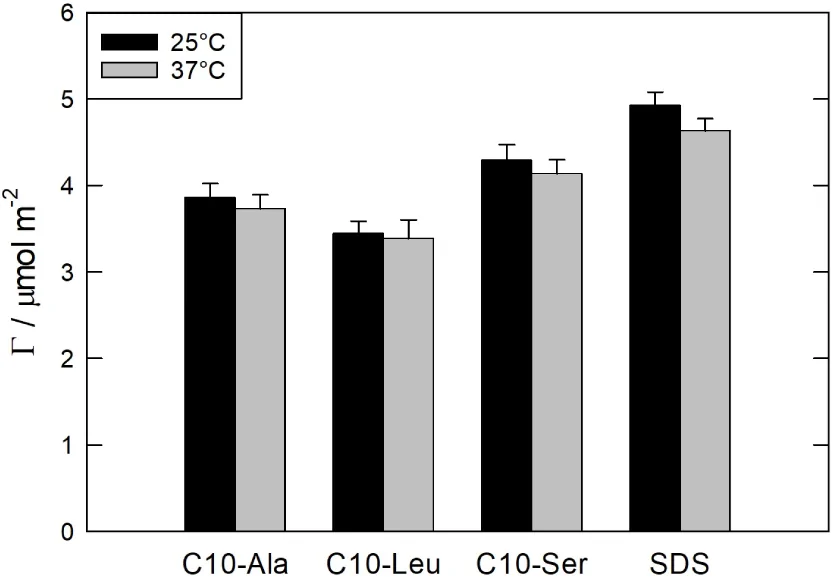
Adsorbed amount Γ at the air/water interface for all surfactants reported as a function of temperature. Results are very close and well within error.

In order to understand the anomaly reported in our previous work^12^ and discuss the interaction between the surfactants studied here and lipid bilayers, we must first introduce the concept of Packing Parameter (PP), which predicts the supramolecular assembly of surfactants from their molecular structure:^29^

6 $$PP=\frac{v}{\mathrm{APM}{\;l}_{c}}$$

Where *v* is the volume of the hydrocarbon chain and *l*_*c*_ the critical chain length, both described as a function of number of carbon atoms (*n*) as

7 $$v=\left(24.4+26.9\;\left(n-1\right)\right)\;{Å}^{3}$$

8 $$l_{c}=\left(1.5+1.26\;\left(n-1\right)\right)\;Å$$

Surfactant with PP between 0.5 and 1 are known to form bilayer structures, while lower PP is typical of cylinders (1/3 < PP < 1/2) or spheres (PP < 1/3). Surfactants with PP > 1 tend to form reverse micelles.^30^

The area per molecule of a DMPC molecule within a lipid bilayer as determined using a combination of SANS and SAXS is between 59.9 and 63.3 Å^2^ (the area occupied by one lipid tail is then ∼30–32 Å^2^),^31^ in excellent agreement with molecular dynamics simulations.^32^ As a result, the PP for DMPC within a lipid bilayer is 0.68 ± 0.02, with a natural tendency to form planar structures.

The packing parameter for the surfactants reported in this study, calculated using eq 6, is shown in Table 3 below. For C_10_-Ala, C_10_-Leu, C_10_-Ser and SDS the APM used was obtained from Neutron Reflectivity measurements, for DMPC the values were obtained from literature.^31^

**Table 3:** Packing Parameter as Determined Using the APM Obtained from Neutron Reflectivity (C_10_-Ala, C_10_-Leu, C_10_-Ser, and SDS) and Data from Literature (DMPC)^31^

Surfactant	Packing parameter (PP)
C_10_-Ala	0.46 ± 0.02
C_10_-Leu	0.42 ± 0.03
C_10_-Ser	0.52 ± 0.02
SDS	0.58 ± 0.02
DMPC	0.68 ± 0.02

C_10_-Leu has an APM, as determined by surface tension data and NR on surfactant monolayers, of around 49.0 ± 3 Å, corresponding to a PP = 0.42 ± 0.03. The insertion of a surfactant with a small PP would introduce curvature to the bilayer structure, leading to destabilization and eventually its collapse. The two surfactants that showed the best interaction profile with the lipid bilayer were C_10_-Ala and C_10_-Ser. They were also those with the smallest area per molecule among those studied with ∼44.5 ± 2 Å and around 40.2 ± 1.5 Å^2^, corresponding to PP = 0.46 ± 0.02 for C_10_-Ala and PP = 0.52 ± 0.02 for C_10_-Ser. Their PPs are closer to the 0.5 threshold; therefore, they are expected to introduce less curvature to the bilayer. This not only explains the bilayer destabilization that only occurred at higher surfactant concentration, but also the higher surfactant volume fraction recorded within the bilayer: more surfactants can accommodate within the lipid bilayer without causing its collapse.^12^

The most likely interpretation is that surfactants with smaller PP increase the curvature of the bilayer more compared to smaller surfactants.^33^ All the C_10_-aa surfactants studied in our previous work were inserting within the bilayer showing comparable profiles up to a concentration around the CMC with fraction ranging from ∼9% for C_10_-Leucine (averaging inner and outer monolayer as the bilayer was asymmetric in nature) and ∼22% for C_10_-Serine.^12^ For C_10_-Leu, however, additional surfactant caused the lipid bilayer to lose its integrity and as observed from parallel SANS measurements, mixed micelles started appearing in solution. For C_10_-Ala and C_10_-Ser, on the other hand, additional surfactant was well tolerated and incorporated within the bilayer up to a very high-volume fraction before bilayer rupture, which occurred respectively at concentration ∼ 3–4·CMC.

Surfactants with larger APM such as C_10_-Leu present a low PP and induce curvature stress upon insertion in the membrane, eventually leading to membrane failure upon reaching a critical concentration. C_10_-Ala has a PP of 0.46 ± 0.02, very close to 0.5 which is the threshold of bilayer structures, while C_10_-Ser has a PP of 0.52 ± 0.02. These surfactants cause less curvature stress upon insertion in the membrane and are therefore much better tolerated, as can be seen both by the highest concentration required for destroying the membrane integrity and the higher surfactant volume fraction.

Clearly, the APM alone cannot fully explain the phenomenon observed as SDS, which produces much more extensive disruption on the lipid bilayer, has an even smaller area per molecule compared to C_10_-Ala and C_10_-Ser. SDS, however, showed a much different behavior compared to the C_10_-surfactants when interacting with a lipid bilayer as measured by NR: the bilayer disruption took place at a concentration lower than that predicted by the SANS measurements. This was attributed to the larger repulsion between the sulfate groups of the SDS molecules and the hydroxyl groups on the silicon oxide surface: as more surfactant is incorporated the repulsion is strong enough to detach the bilayer from the surface. Notably, SDS has a small, strongly hydrated sulfate headgroup with a permanent charge, while C_10_-amino acid surfactants have bulkier head groups, with different hydration and, possibly, different molecular geometry. The comparison with SDS indicates that while interfacial packing, as reflected by the APM and PP, provides valuable information on molecular organization at interfaces, membrane disruption may be harder to predict from these descriptors alone. The chemistry and hydration of the surfactant headgroup, together with the energetics of partitioning into the lipid bilayer, must also be considered.

## Conclusions

^12^

In this work, neutron reflectivity was used to quantitatively determine the APM and Γ of selected C_10_-based amino acid surfactants at the air/buffer interface, providing insight into their interfacial packing and molecular organization. These parameters were compared with values derived from surface tension measurements to complement our previous investigation of the interactions of the same surfactants with phospholipid bilayers.^12^

Neutron reflectivity showed that all three C_10_ amino acid surfactants form monolayers of similar thickness (∼23.5 Å), slightly more disordered than SDS, despite their shorter alkyl chains. Among the series, C_10_-Leu exhibited the largest APM, which is consistent with the combined influence of its bulkier and more hydrophobic headgroup on interfacial packing. In contrast, C_10_-Ser displayed the smallest APM, likely reflecting the ability of its hydroxyl-containing side chain to promote favorable orientation, hydration, and hydrogen-bonding interactions that facilitate more compact molecular packing. Importantly, the APM values derived from NR agreed well with those obtained from surface tension for C_10_-Ser and lay within the uncertainty of the surface tension measurements for C_10_-Leu and C_10_-Ala, highlighting the greater sensitivity of NR for resolving subtle differences in interfacial packing.

The interfacial parameters determined in the present study provide the physicochemical basis for interpreting the differences in membrane interactions previously observed for this homologous surfactant series. Surfactants with smaller APM and higher packing density, such as C_10_-Ser, are incorporated into lipid bilayers to a greater extent and remain accommodated above the CMC, whereas surfactants with larger effective headgroups, such as C_10_-Leu, exhibit reduced bilayer incorporation and induce membrane disruption at lower surfactant concentrations. Although the air/water interface is not intended to represent a phospholipid bilayer directly, it provides a well-defined model system for comparing the intrinsic packing characteristics of closely related surfactants under identical experimental conditions. The intermolecular interactions governing adsorption at the air/water interface differ from those operating within lipid membranes, where lipid headgroups, hydration, and specific surfactant–lipid interactions also contribute to the free energy of insertion. Consequently, the area per molecule and packing parameter should be regarded as descriptors of intrinsic molecular packing tendencies rather than sole predictors of membrane activity. Nevertheless, the clear correlations observed across the C_10_ amino acid surfactant series indicate that these structural parameters provide a useful framework for rationalizing the relative ability of structurally related surfactants to be accommodated within planar lipid bilayers before membrane disruption occurs.

Finally, the negligible differences observed between measurements performed at 25 and 37 °C indicate that the adsorption behavior of these surfactants is largely unaffected over this temperature range, including physiological temperature. Overall, this work provides a comprehensive interfacial characterization of C_10_ amino acid surfactants by neutron reflectivity and demonstrates a connection between intrinsic interfacial packing with membrane interactions. These findings contribute to a more complete understanding of this class of amino acid surfactants and support their rational development for pharmaceutical and biomedical applications.

## Supplementary Material



## Notes

Supporting Information: surface tension of h-SDS solutions in isotonic phosphate buffer at 25 °C (Figure S1), adsorbed amount (Γ) as determined from neutron reflectivity for the surfactants studied at all concentrations (Table S1) (PDF)
