# Supplementary material for: Adsorption Behavior of C10 Amino Acid Surfactants at the Air/Water Interface: Implications for Membrane Compatibility
Source: Langmuir. 2026 Sep 21;42(39):28756–65. doi: 10.1021/acs.langmuir.6c02078 (PMC13648267; doi:10.1021/acs.langmuir.6c02078)
Supplement: Supplementary file 1 [file la6c02078_si_001.pdf]

Supporting information

Adsorption behaviour of C_{10} amino acid surfactants at air/water interface: Implications for membrane compatibility

Diego R. Perinelli[1], Michele Verboni[2], Mattia Tiboni[2], Kun Ma[3], Peixun Li[3], Giulia Bonacucina[1], Marco Cespi[1], Simone Lucarini[2], Andrea Duranti[2], Giovanni Filippo Palmieri[1], Mario Campana[3]* and Luca Casettari[2]*

[1]Chemistry Interdisciplinary Project (ChIP), School of Pharmacy, University of Camerino, 62032, Camerino, MC, Italy

[2]Department of Biomolecular Sciences, School of Pharmacy, University of Urbino Carlo Bo, Via Ca le Suore 2, Urbino, PU 61029, Italy

[3]ISIS Pulsed Neutron & Muon Source, STFC Rutherford Appleton Laboratory, Didcot OX110QX, UK

*Corresponding authors: Diego Romano Perinelli (diego.perinelli@unicam.it), Mario Campana (mario.campana@stfc.ac.uk)

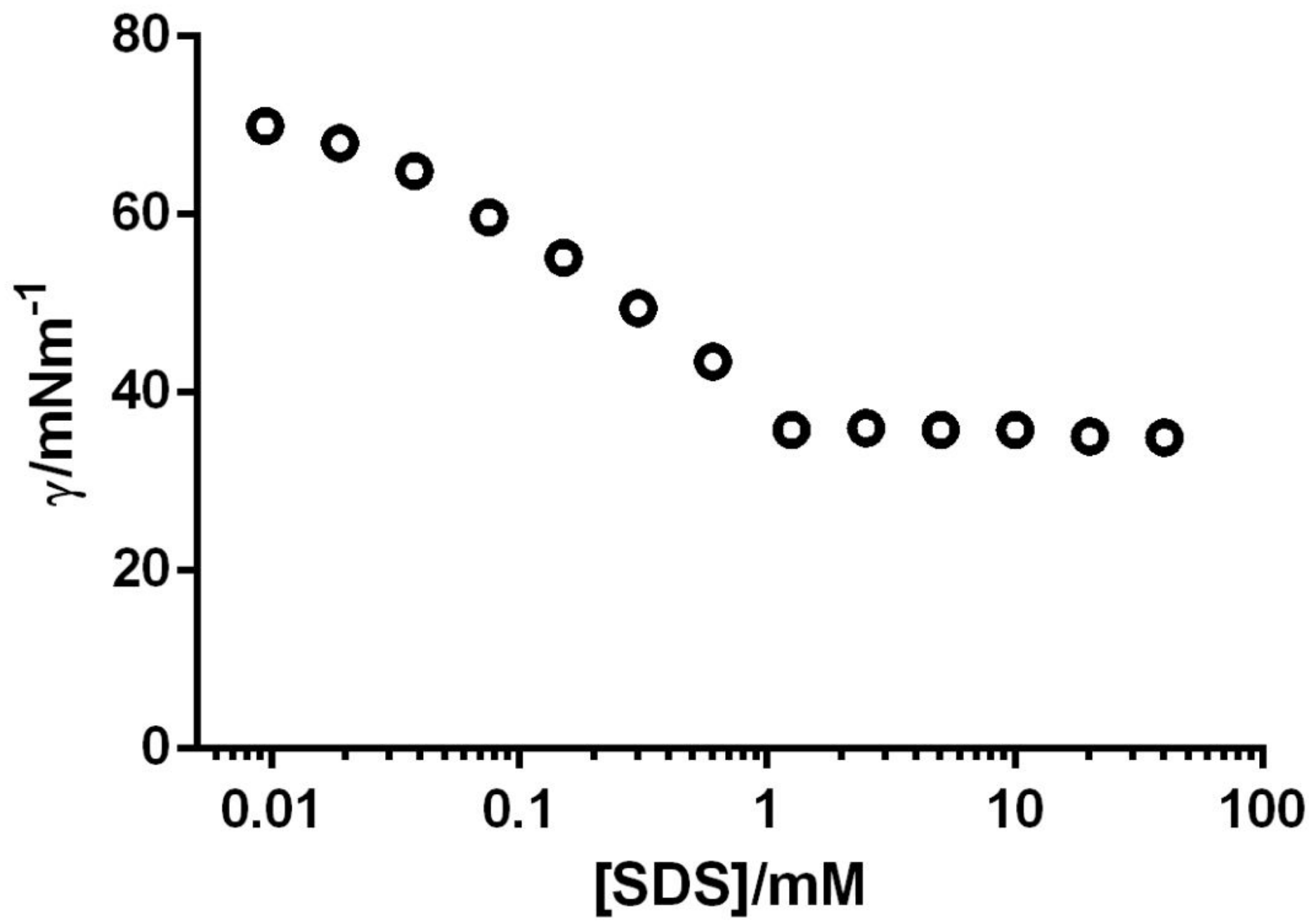

Figure S1. Surface tension of h-SDS solutions in isotonic phosphate buffer at 25˚C

Table S1. Adsorbed amount (Γ) as determined from neutron reflectivity for the surfactants studied at all concentrations.

[C_{10}-Leu] / mM	Adsorbed amount Γ / μmol m^{-2} Min	Optimum	Max
20	3.31	3.44	3.59
15	3.30	3.42	3.57
8.82	3.20	3.35	3.50
3.63	2.95	3.08	3.22
1.1	2.87	3.08	3.32
0.5	2.51	2.68	2.86
0.1	1.78	1.92	2.08

[C_{10}-Ala] / mM	Adsorbed amount Γ / μmol m^{-2} Min	Optimum	Max
20	3.71	3.86	4.02
12.8	3.46	3.59	3.75
6	2.96	3.11	3.27
3.84	2.57	2.73	2.91
0.6	1.78	1.91	2.05

[C_{10}-Ser] / mM	Adsorbed amount Γ / μmol m^{-2} Min	Optimum	Max
20	4.12	4.29	4.47
12	4.18	4.34	4.54
6	3.57	3.74	3.93
3.12	3.39	3.50	3.65
1.5	2.71	2.86	3.01

[SDS] / mM	Adsorbed amount Γ / μmol m^{-2} Min	Optimum	Max
5	4.78	4.92	5.08
2	4.74	4.89	5.07
0.56	4.27	4.40	4.56
0.15	3.28	3.40	3.53
0.06	2.57	2.73	2.92
